# Evaluation of anxiolytic activity of the essential oil of the aerial part of *Foeniculum vulgare* Miller in mice

**DOI:** 10.1186/1472-6882-14-310

**Published:** 2014-08-23

**Authors:** Miraf Mesfin, Kaleab Asres, Workineh Shibeshi

**Affiliations:** Department of Pharmacology and clinical Pharmacy, School of Pharmacy, Addis Ababa University, Addis Ababa, Ethiopia; Department of Pharmaceutical Chemistry and Pharmacognosy, School of Pharmacy, Addis Ababa University, Addis Ababa, Ethiopia

**Keywords:** Anxiolytic activity, *Foeniculum vulgare*, Essential oil, Mice

## Abstract

**Background:**

*Foeniculum vulgare* locally known as *ensilal*, is an aromatic plant widely cultivated in temperate and tropical regions. The anti-anxiety activity of the crude extract of F*. vulgare* has been reported. However, the fraction responsible for anxiolytic activity is not known and there is no any report on the anti-anxiety activity of the essential oil of *F. vulgare.* The objective of study was to evaluate the anxiolytic activity of the essential oil of *Foeniculum vulgare* Miller.

**Methods:**

Adult Swiss albino male mice were randomly divided into six groups (n = 6). Groups I and II received Tween 80 (5%, v/v) and diazepam (0.5 mg/kg, ip), respectively, while groups III to V received orally 50, 100, and 200 and 400 mg/kg doses of the essential oil of *F. vulgare*, respectively. The mice were then individually placed in animal anxiety models: elevated plus maze (EPM), staircase test (SCT) and open field test (OFT) and evaluated for various parameters.

**Results:**

In EPM test, 100 and 200 mg/kg doses of the essential oil significantly increased percent number of entries and time spent in open arms compared to control. In SCT these doses also reduced rearing significantly compared to controls, while only the 200 mg/kg dose significantly increased number of squares crossed at the center in the OFT test.

**Conclusion:**

The essential oil of *F. vulgare* was found to exhibit a promising anxiolytic activity.

## Background

Anxiety disorders are among the most common mental, emotional, and behavioral problems affecting one-eighth of the total population worldwide, and have become a very important area of research interest in psychopharmacology. Anxiety represents a heterogenous group of disorders, probably with no single unifying etiology; various psychodynamic, psychoanalytic, behavioral, cognitive, genetic and biological theories have been proposed to explain the etiology of anxiety disorders [1]. It is reported to have increasing prevalence in recent cohorts in many countries and to have much earlier ages of onset than other commonly occurring chronic conditions [2]. Anxiety disorders cause performance impairments on numerous tasks and are associated with high rates of medically unexplained symptoms, increased utilization of healthcare, strongly and independently associated with chronic medical illnesses, low levels of quality of life and disability [3, 4]. Pharmacologic treatment of anxiety through the ages has included different drugs. The first class of drugs developed (barbiturates) was highly effective, unfortunately, the barbiturates can cause respiratory arrest and have a narrow therapeutic index [5]. The benzodiazepines were developed as a safer alternative to barbiturates, however, their beneficial effects are overshadowed by the emergence of physical and psychological dependence and withdrawal reactions [6, 7]. Other drugs used for treatment of anxiety having unfavorable side-effect profiles include buspirone [8, 9], antidepressants [10–12] and beta-blockers [5].

Due to adverse effects associated with the currently available drugs, patients on anxiolytic drugs usually terminate the treatment before full recovery [13]. In addition, one-third of patients in controlled studies are unresponsive to any one of the medications [14]. Thus, there is a critical need for development of newer anxiolytic agents. In the search for new therapeutic products for the treatment of neurological disorders, medicinal plant research, worldwide, has progressed constantly, demonstrating the pharmacological effectiveness of different plant species in a variety of animal models [15]. Several essential oils obtained from plants are employed in order to balance emotions, improve physical and mental well-being [16]. *Foeniculum vulgare* Mill. locally known as ensilal or commonly known as fennel, is an aromatic plant widely cultivated in temperate and tropical regions [17].

The chemical constituents of *F. vulgare* include essential oil, fatty acid, phenylpropanoids, monoter-penids, sesquiterpenes and coumarins. It also contains triterpenoids, tannins, flavonoids, cardiac glycosides, saponins, and other types of compounds [18, 19].

The essential oil of *F. vulgare* is characterized by the presence of several components including major components trans-anethole, fenchone, methyl chavicol, eugnol, limonene, *p*-anisaldehyde, α-phellandrene, α-pinene, 1,8-cineole, γ-terpinene and *P*-cymene. Compounds such as 3-methylbutanol, linalool, β-pinene, mycerene, δ-3-carene, camphor, α-terpinol, cis-anethole, thymol are also found in lower concentration [20]. The essential oil of *F. vulgare* is generally recognized safe as the toxicity it may cause is negligible [21].

In traditional medicine, *F. vulgare* is used to: encourage menstruation and lactation, stimulate gastrointestinal motility, relieve intestinal gas accumulation, improve eyesight, alleviate productive coughs, ease spasm, promote courage and mental strength, reduce stress and nervousness and produce calming [22, 23]. The medicinal properties of *F. vulgare* as evidenced by different animal and clinical studies include, antibacterial and antifungal [24], antioxidant [25], anti-inflammatory [26], anti-atherosclerotic [27], gastroprotective [28], hepatoprotective [29], and diuretic [30]. The anti-anxiety activity of the crude extract of F*. vulgare* has been reported [31, 32]. However, the fraction responsible for anxiolytic activity is not known and there is no any report on the anti-anxiety activity of the essential oil of *F. vulgare*.

Thus, the objective of this study was to evaluate the anxiolytic activities of essential oil of *F. vulgare* in animal models of anxiety.

## Methods

### Chemicals

The chemicals used in the experiment include diazepam (Intlas Pharmaceuticals, India), tween 80 (Research-lab fine Chem Industries, India), anhydrous sodium sulfate (Bio-lab laboratories Ltd, Israel) and ethyl alcohol (Changshu Yangyuan Chemical, China).

### Plant material

The fresh aerial parts of *F. vulgare* were purchased from local market in Addis Ababa. The plant was identified at the National Herbarium, College of Natural Sciences, Addis Ababa University, where a voucher specimen was deposited (collection number MM001).

### Experimental animals

Adult Swiss albino male mice weighting 25-35 g were obtained from rodent breeding unit of the School of Pharmacy, Addis Ababa University. The animals were housed under standard environmental conditions and were allowed free access to tap water and standard laboratory pellet *ad libitum*. The ethical handling (33) of mice used in our study and the experimental protocols used were approved by Research and Ethics committee of the School of Pharmacy, Addis Ababa University.

### Extraction of essential oil

About 3 Kg of the fresh leaves of *F. vulgare* were distilled by hydrodistillation for a period of about 3 h using Clevenger-type apparatus. The oil was dried over anhydrous sodium sulphate to remove traces of moisture and stored in a vial inside a refrigerator at 4°C until use.

### Acute toxicity study

Acute oral toxicity study was determined by using five female mice weighing 25–30 g. The mice were fasted for 3 h prior to the experiment. The mice were administered using oral gavage a single high dose (2000 mg/kg) of the essential oil and were observed for any symptoms of toxicity continuously for 1 h, intermittently for 4 h, over a period of 24 h and for 14 days [33].

### Experimental designs

The mice were randomly divided into six groups (n = 6). The first group received the vehicle (5% Tween 80 in distilled water), the second group received standard drug, diazepam (0.5 mg/kg, Ip). Groups III to VI using oral gavage received 50, 100, 200 and 400 mg/kg doses of the essential oil, respectively. The doses were selected based on the results of the oral acute toxicity study. Taking into account the safety of the essential oil, 1/10th of the maximum dose (2000 mg/kg) given in the toxicity study limit test was considered as a middle dose (200 mg/kg). Double, one-fourth and half of the middle dose were assigned as high dose (400 mg/kg), first low dose (50 mg/kg) and second low dose (100 mg/kg) respectively.

After 30 min of diazepam treatment or 60 min essential oil/vehicle pretreatment, the mice were individually placed in animal models. All the tests were carried out at night with a minimal amount of background noise. After each test, the maze was cleaned with ethyl alcohol to eliminate any olfactory cues to the next animal [34].

### Elevated plus maze (EPM)

The test was conducted using apparatus validated by Lister [35]. After each mouse was placed in the centre of the maze facing one of the open arms, the number of entries made into the open and closed arms and the time spent in them was recorded, using a video camera for the next five min. From these data, the percentage of entries and the percentage of time spent in each arms was calculated.

### Staircase test (SCT)

SCT was carried out as used by Simiand et al*.* [36]. Each mouse was placed on the floor of the box with its back to the staircase, then the number of steps climbed and the numbers of rears were recorded over 3 min period using a video camera placed over head. A step is considered to be climbed only if the mouse had placed all four paws on the step, the number of steps descended was not taken into account.

### Open field test (OFT)

The test was conducted as described by Aragao et al. [37]. Briefly, the mice were placed individually in a corner square of the OFT apparatus and the total number of squares crossed at the periphery, number of central squares crossed, total number of squares traveled, and the time the mouse spent in the center were video recorded for 5 min.

### Statistical analysis

The data are presented as mean ± S.E.M (standard error of mean) of six mice. Statistical analysis of the data was performed using one-way analysis of variance (ANOVA) followed by Tukey posthoc test. Significant differences were set at P values lower than 0.05.

## Results

### Acute toxicity test

After the administration of 2000 mg/Kg dose of the essential oil of *F. vulgare,* the animals didn’t show loss of weight, autonomic behavioral changes or other signs of toxicity. There was also no mortality observed in the study period, suggesting that the LD50 (median lethal oral dose) of the essential oil is higher than 2000 mg/kg when given orally [33].

### Anxiolytic effects

**Figure 1:**
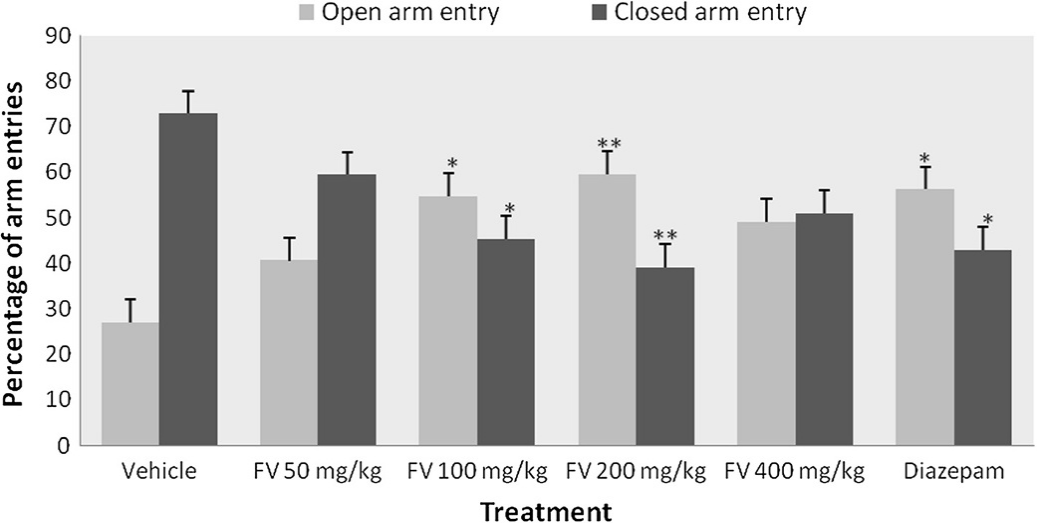
**Effects of the essential oil of** ***Foeniculum vulgare*** **and controls on percentage of arm entries in the elevated plus maze (EPM) test in mice.** Data are mean ± S.E.M (n = 6); *P < 0.05, **P < 0.01, compared with vehicle. FV = *Foeniculum vulgare* essential oil.

**Figure 2:**
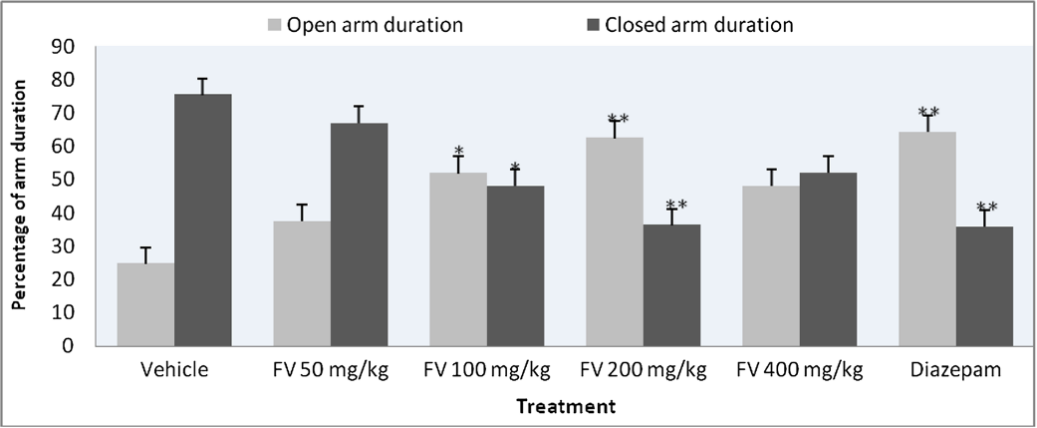
**Effects of the essential oil of** ***Foeniculum vulgare*** **and controls on the percentage of duration in arms in the elevated plus maze test in mice.** Data are expressed as mean ± S.E.M. (n = 6); *P < 0.05, **P < 0.01 as compared to control. FV = *Foeniculum vulgare* essential oil.

In the EPM paradigm (Table 1), the essential oil at 200 mg/kg dose showed significant increase in the number of entries into the open arms compared to the negative control, while all other doses of the oil did not significantly affect the parameter compared. On the other hand, none of the doses of the oil or diazepam significantly affected the number of closed arm entries except the 400 mg/kg dose which significantly decreased the parameter. The time the mice spent in the open arms was significantly increased in animals treated with 100 and 200 mg/kg doses of the oil compared to the control group. Mice treated with 100 and 200 mg/kg doses significantly increased the percentage of open arm entry compared to the vehicle (Figure 1) and at a dose of 200 mg/kg the oil produced a significant reduction in the percentage of time spent in the closed arms (Figure 2).

**Table 1:** **The behavior of mice treated with the essential oil of** ***Foeniculum vulgare*** **and reference compounds in the elevated plus maze model**

Treatment	Dose, mg/kg	Number of entries (counts/5 min)			Time spent (sec/5 min)		
		Open arm	Closed arm	Total	Open arm	Closed arm	Center
Vehicle	-	3.17 ± 1.33	8.50 ± 2.59	11.67 ± 3.66	44.17 ± 20.19	137.83 ± 38.24	118.00 ± 41.25
FV	50	4.83 ± 1.72	6.83 ± 0.75	11.66 ± 1.37	68.00 ± 25.69	117.50 ± 46.78	114.50 ± 50.03
FV	100	7.50 ± 4.59	5.83 ± 3.31	13.66 ± 3.78	109.67 ± 48.64^a*^	99.83 ± 43.32	90.50 ± 33.59
FV	200	11.00 ± 5.48^a*^	8.00 ± 4.90	19.00 ± 9.87^c*^	112.67 ± 35.62^a*^	71.33 ± 37.17^a*^	119.00 ± 36.87
FV	400	4.01 ± 2.10	4.00 ± 1.4 ^a^^*^	8.00 ± 2.76^(b, d)*^	87.50 ± 36.32	93.33 ± 35.27	119.17 ± 27.13
Diazepam	0.5	10.83 ± 9.06^a*^	6.50 ± 2.59	17.50 ± 8.53 ^c*^	123.67 ± 50.67^a**^	67.33 ± 34.63^a*^	84.67 ± 44.23

Data are presented as mean ± S.E.M, n = 6, *P < 0.05, **P < 0.01, ^a^against control, ^b^against 200 mg/kg, ^c^against 400 mg/kg, ^d^against diazepam; FV = *Foeniculum vulgare* essential oil.

In SCT (Table 2), mice treated with the 100 and 200 mg/kg doses of the oil showed significant reduction of rearing compared to control group, however, only 200 mg/kg dose of the oil showed statistically significant reduction in rearing compared with diazepam. The reduction in the number of climbing was produced by the essential oil only at 400 mg/kg dose compared with control, while other doses were not effective.

**Table 2:** **Effect produced by administration of the essential oil of** ***Foeniculum vulgare*** **and reference compounds to mice in the staircase test**

Treatment	Dose (mg/kg)	Number of rearing	Number of stairs climbed
Vehicle	-	19.67 ± 2.50	16.17 ± 6.11
FV	50	15.00 ± 1.67 ^d**^	18.00 ± 4.19 ^e**^
FV	100	13.33 ± 4.93^a* d*^	13.34 ± 1.37
FV	200	8.00 ± 3.03 ^(a, b, e)**. (c, f)*^	11.50 ± 3.56
FV	400	18.33 ± 3.44 ^d**^	9.50 ± 4.09 ^a*. b**^
Diazepam	0.5	14.00 ± 3.29^(a*.d*)^	12.00 ± 4.56

Data are mean ± S.E.M, n = 6, ^a^compared with vehicle, ^b^to 50 mg/kg, ^c^to 100 mg/kg, ^d^to 200 mg/kg, ^e^to 400 mg/kg, ^**f**^to diazepam, *P < 0.05, **P < 0.01. FV = *Foeniculum vulgare* essential oil.

In the OFT model, the number of central squares crossed was increased in all groups treated with the essential oil, however, statistical significance was reached only in the groups treated with 200 mg/kg compared to the control (Table 3). In mice treated with 400 mg/kg the oil, a significant reduction in the number of squares crossed at the periphery compared to both vehicle and 200 mg/kg dose was noted. Mice treated with 100 and 200 mg/kg dose of the essential oil spent more time in the central squares compared to the control group.

**Table 3:** **Effect of the essential oil of** ***Foeniculum vulgare*** **and reference compounds on the behavior of mice in the open field model**

Treatment	Time spent in central squares (sec)	Number of squared crossed		
		Center	Periphery	Total
Vehicle	2.17 ± 1.33	2.00 ± 1.26	85.17 ± 19.37	87.16 ± 19.31
FV50	3.33 ± 5.92 ^c*^	3.67 ± 7.12 ^d*^	80.83 ± 25.34	84.50 ± 25.99
FV100	10.67 ± 4.32 ^(a, b)*^	9.00 ± 7.54	79.33 ± 10.32 ^f**^	88.33 ± 17.81 ^f**^
FV200	10.50 ± 4.04 ^a*^	14.50 ± 3.27 ^(a,b,e)*^	87.50 ± 19.67 ^(e, f)*^	102.00 ± 21.87 ^e*^
FV400	4.50 ± 5.92	3.34 ± 4.55 ^d*^	53.00 ± 4.94 ^(a ,d)*^	56.33 ± 7.76 ^d*^
Diazepam	9.33 ± 5.32 ^a*^	12.83 ± 10.66 ^a*^	122.67 ± 31.6^(a, d)*.c**^	133.83 ± 34.93^(a, c)**^

The data are mean ± S.E.M, n = 6, ^a^against control, ^b^against 50 mg/kg, ^c^against 100 mg/kg, ^d^against 200 mg/kg, ^e^against 400 mg/kg, ^f^against diazepam, *P < 0.05 and **P < 0.01. FV = *Foeniculum vulgare* essential oil.

## Discussion

The present work has evaluated the anxiolytic activity of various doses of the essential oil of *F. vulgare* in mice employing three non-conditioned behavioral animal models of anxiety; EPM model, OFT and SCT. These tests are classic and standard models for screening central nervous system actions providing information about anxiety and psychomotor performance [38]. Further, these models can create an anxiety state in normal rodents in a reproducible paradigm while minimizing some of the confounding factors of other conditioned assays [39].

The EPM test is principally based on the behavior that exposure of animals to an elevated maze alley evokes an approach-avoidance conflict and rodents consistently spend greater time in the closed arms when placed in mazes comprising of open and closed arms [40]. Based on these assertions, EPM tests are reliable means of identifying selective anxiolytic effect of drugs and used as a tool in the investigation of the psychological and neuro-chemical basis of anxiety, for screening anxiety-modulating drugs or mouse genotypes [41]. The EPM test has been validated pharmacologically, physiologically and behaviorally, and has become one of the most widely used behavioral tests for anxiety [34]. Anxiety is induced by a fear due to height in rodents when placed on the EPM. The ultimate manifestation of anxiety is exhibited by preference to remain at safer places and a decrease in the motor activity.

Treatment of mice with the essential oil of *F. vulgare* resulted in significant alterations on the behavioral responses measured in the EPM test. Experimental animals treated with 100 and 200 mg/kg doses of *F. vulgare* essential oil showed significantly increased percentage of number of entries into the open arms. This decreased aversion to open arms compared to control group indicated its anxiolytic activity [40]. The decrease in aversion to open arms by the 100 and 200 mg/kg doses of the oil was also verified by the increased percentage of time spent in the open arms compared with the negative control.

Time spent in the central platform of EPM appears to be related to decision making and/or risk assessment. A decreased time spent on the central platform serves as indicator of a reduced decision making behavior, a parameter accepted as reliable indicators of anxiety and fearfulness [42]. However, neither doses of the essential oil nor diazepam altered the parameter significantly compared to control. At a dose of 400 mg/kg, however, the oil showed a significant decrease on the number of entries in the closed arms. The absence of significant modification in the number of closed arm entries, in the anxiolytic doses of the oil, in the EPM indicated that the anxiolytic activity was observed at doses that did not impair motor activity [43].

The EPM test is one of the most popular tests for search of new benzodiazepine-like anxiolytic agents [44]. In this context, the activity of the essential oil of *F. vulgare* (100 and 200 mg/kg) in relieving anxiety in this model may suggest a possible positive modulation of the GABA-A/benzodiazepine receptor complex.

The SCT for screening anxiolytic activity is a simple and rapid procedure for preliminary screening of anxiolytic agents [45]. Step climbing reflects exploratory or locomotor activity, while rearing behavior was a manifestation of anxiety state [46]. The present study showed that exposure of the experimental animals to the essential oil of *F. vulgare* significantly reduced rearing activity at doses (100 and 200 mg/kg) that did not suppress climbing, aligning with a behavior of an anxiolytic compound in a staircase model [47]. At a dose of 400 mg/kg, the essential oil produced a significant reduction in the number of steps ascended indicating suppression of locomotor activity, which is interpreted as a sedative, rather than anxiolytic effect in different studies [46, 48]. Only GABA receptor complex active agents have been shown to reduce rearing at doses that do not reduce climbing in the SCT. Other non-benzodiazepine compounds induce non-specific suppression of both rearing and climbing behavior [49], strengthening the suggested possible mechanism of the oil.

The OFT is a classical animal model used to evaluate effects of drugs on anxiety, general motor activity and exploratory behavior. It uses the normal aversion of rodents to an open, brightly lit area, as the confrontation with the situation induces anxiety behavior in rodents [50]. Rodents normally spend more time in the protective corners, suggesting that the walls confer anxiety-relieving body contact. When animals are placed in OFT, they express their anxiety by a decrease in exploratory behavior [51].

Increased entry and the time spent in the center of the arena in the OFT reflects decreased anxiety altered by anxiolytic property, as measures of central exploration are often regarded as anxiety-related indices [52]. Diazepam significantly increased the total number of squares traveled at the periphery, which may be an increase in exploratory activity on reduced anxiety. The anxiolytic activity of the essential oil at the effective doses (100 and 200 mg/kg), could not be confirmed by this parameter, as the increase in locomotor activity can be used as indice of anxiolytic effect [53]. The decrease in ambulation at the highest dose (400 mg/kg) of the oil might be due to the sedative property of the oil. Suppression of exploratory behavior is an indication of central nervous system depressant activity [44]. This decline in activity at highest doses of the essential oil in EPM, SCT and OFT can be correlated well with each other.

In the present study, the activity of *F. vulgare* corresponding to the doses given had seemingly a biphasic inverted-U (bell) shape, as the administration of the highest (400 mg/kg) and lowest (50 mg/kg) doses showed insignificant low effects whereas the 100 mg/kg and 200 mg/kg dose levels produced significant effects and the peak in mean response was shown at 200 mg/kg. The ineffectiveness of the lowest dose of the oil may be because it is a sub-threshold level. However, the lack of effect at the highest dose level may be due to a decreased locomotion which might be due to an interference with a normal sensory-motor function or sedative effect. The sedative activity of the essential oil is also verified by a research [54]. Such inverted U-shaped functions are also typical of conventional anxiolytics (benzodiazepines and buspirone) that, in addition to reducing anxiety, also impair motor performance at high doses [55].

The effects of *F. vulgare* essential oil in the anxiety models were comparable or better in some cases to those of the standard anxiolytic drug diazepam at the doses used. Overall, the results indicated that the essential oil of F. vulgare can be further studied for optimum dosage to be used as a future of anti-anxiety drug development or as a standardized Phytomedicine.

The major component of the essential oil, anethole, which is the methyl ether of estrone, has been reported to display a potent estrogenic activity [56]. There is a correlation between decreased estrogen levels and increased anxiety [57, 58]. Further, anethole has a structural similarity with dopamine [59]. In other studies [60, 61], the minor components of the essential oil of *F. vulgare* including (+)-limonene, linalool, pinene and eugenol have also been reported to have anxiety relieving like activities.

## Conclusion

The lower doses of the essential oil of the aerial parts of *F. vulgare* possess anxiolytic activity, while at a higher dose the oil appears to be potentially sedative. Thus, the results of the present study indicate that the essential oil of *F. vulgare* may have potential clinical applications in the management of anxiety.

## Abbreviations

F. vulgare: Foeniculum vulgare
EPM: Elevated plus maze
SCT: Staircase test
OFT: Open field test.
